# Reward, motivation and brain imaging in human healthy participants – A narrative review

**DOI:** 10.3389/fnbeh.2023.1123733

**Published:** 2023-03-24

**Authors:** Aviv M. Weinstein

**Affiliations:** Department of Psychology and Behavioral Sciences, Ariel University, Ari’el, Israel

**Keywords:** brain imaging, dopamine, memory, motivation, reward

## Abstract

Over the past 20 years there has been an increasing number of brain imaging studies on the mechanisms underlying reward motivation in humans. This narrative review describes studies on the neural mechanisms associated with reward motivation and their relationships with cognitive function in healthy human participants. The brain’s meso-limbic dopamine reward circuitry in humans is known to control reward-motivated behavior in humans. The medial and lateral Pre-Frontal Cortex (PFC) integrate motivation and cognitive control during decision-making and the dorsolateral PFC (dlPFC) integrates and transmits signals of reward to the mesolimbic and meso-cortical dopamine circuits and initiates motivated behavior. The thalamus and insula influence incentive processing in humans and the motor system plays a role in response to action control. There are reciprocal relationships between reward motivation, learning, memory, imagery, working memory, and attention. The most common method of assessing reward motivation is the monetary incentive delay task (DMRT) and there are several meta-analyses of this paradigm. Genetics modulates motivation reward, and dopamine provides the basis for the interaction between motivational and cognitive control. There is some evidence that male adolescents take more risky decisions than female adolescents and that the lateralization of reward-related DA release in the ventral striatum is confined to men. These studies have implications for our understanding of natural reward and psychiatric conditions like addiction, depression and ADHD. Furthermore, the association between reward and memory can help develop treatment techniques for drug addiction that interfere with consolidation of memory. Finally, there is a lack of research on reward motivation, genetics and sex differences and this can improve our understanding of the relationships between reward, motivation and the brain.

## 1. Introduction

There has been a significant growth in research on the brain mechanisms mediating reward motivation in humans. The purpose of this narrative review is to summarize studies on the neural mechanisms associated with reward motivation and cognitive function in healthy human participants from 2000 until now. A PubMed search has used “Reward motivation and brain imaging” as search words for publications between 2000 and October 2022. There were 2,127 records which were screened and papers that were published in English language in peer-reviewed journals and measured brain imaging in human subjects were included. Based on these criteria, 2,058 records were excluded, and 64 records were included.

The brain’s meso-limbic dopamine reward circuitry is known to control reward-motivated behavior in humans. In this review, in the first section, we have described first the brain’s meso-limbic dopamine reward circuitry (1.1) and then its interactions with decision-making (1.2), anticipation of reward (1.3), the coding of reward in the human brain (1.4), motivated cognitive control (1.5), prefrontal cortex (PFC) integration of cognitive control (1.6), reward motivation of the motor system (1.7) and finally, how reward motivation affects incentives like food, money and social reward (1.8). In the following section we have discussed how reward motivation is associated with cognitive processing such as learning (2.1), memory and dopaminergic activity in the striatum (2.2), Memory formation (2.3) Memory encoding and consolidation (2.4) Memory recollection (2.5) Long-term memory of novelty (2.6) Episodic memory retrieval (2.7), motor imagery and imagery of future events (2.8), working memory (2.9) and attention (2.10). Finally, we discuss the most common method of assessing reward motivation, the monetary incentive delay task (DMRT) and the meta-analyses of this paradigm (3.1), implications for drug abuse mental and neurological disorders (3.2) we summarize motivation reward, learning, memory and attention (3.3), motivation reward, pharmacological effects and genetics of dopamine (3.4), reward motivation and sex differences (3.5), and our conclusions (3.6).

### 1.1. The brain’s meso-limbic dopamine reward circuitry

The brain’s meso-limbic dopamine reward circuit is known to control reward-motivated behavior in humans. Schott et al. (2008) have measured neural activity in the meso-limbic reward circuit in participants who performed on a delayed monetary incentive task in fMRI while measuring dopamine (DA) release by using [^11^C]raclopride in PET. They have shown that neural activity in areas responsible for dopamine neurotransmission (substantia nigra and ventral tegmental area SN/VTA), and DA release (ventral striatum and nucleus accumbens VS/NAcc) in fMRI correlated with DA release in the NAcc measured in PET (Schott et al., 2008). Furthermore, DA release correlated with activity in the amygdala and the hippocampus. This study combined neurochemical and magnetic imaging in order to study reward motivation emotion and cognitive function. Kasanova et al. (2017) have measured dopamine receptors D_2_ and D_3_ occupancy with [^18^F]fallypride in PET while participants performed on a probabilistic reinforcement learning task with daily life reward. They have reported an association between reward-related DA release in the putamen, caudate and the VS and task performance. Furthermore, DA release in the right caudate and VS have regulated pleasurable behavior. This study demonstrates the relationships between DA activity, motivation and rewarding behavior. Taken altogether, the combination of measuring dopamine release in the striatum while monitoring brain activity during natural reward improves our understanding of the neurochemical and neuronal mechanisms underlying reward motivation, emotion and cognition in the human brain.

### 1.2. Decision-making

The process of decision-making involves various stages such as a selection phase and an anticipation phase. The wheel of fortune task with monetary gains was used to assess decision-making in fMRI (Ernst et al., 2004). The selection phase has employed visual-spatial attention by activating the occipital-parietal pathway, conflict resolution by activating the ACC, quantity manipulations by activating the parietal cortex and preparation for action by the pre-motor area. The anticipation phase involved reward processing in the VS. The VS was activated during high-reward and risk conditions during selection but not during anticipation. Selection of high-risk and reward and anticipation has activated the OFC. These findings imply that different neural networks are involved during the anticipation and selection phases while performing on a monetary reward task, and that selection and anticipation of risky decision affect VS and the OFC differently.

### 1.3. Anticipation of reward

Anticipation of reward is an important skill in a constantly changing environment and decision-making requires an evaluation of anticipated outcomes and future actions. Symmonds et al. (2010) have used a sequential choice task in order to investigate models of decision valuation and choice of strategy in fMRI. They have reported that participants have evaluated decisions and updated strategies during assessment of actions. Sequential choice was associated with monitoring of future reward. Although the anticipation of reward involves both dorsal and ventral striatal circuits, only the dorsal striatum and its connected cortical network is involved in the direct modulation of motor behavior by incentive motivation. Harsay et al. (2011) have used a reward-cued anti-saccade paradigm to investigate how motivational goals modulate patterns of neural activation and functional connectivity to improve preparation for performance. They have found that reward anticipation was associated with increased activation in the ventral and dorsal striatum, and cortical oculomotor regions.

Potential rewards have an incentive motivation for improving performance. Vassena et al. (2014) have used a mental arithmetic task with manipulations of in fMRI. They have found that a difficult task and high reward have activated the striatum and ACC confirming their role in motivated reward. Reward anticipation is important for directing behavior toward stimuli with positive valence in order to form motivational salience. Mori et al. (2019) have used a monetary incentive delay task gain in order to investigate reward anticipation in fMRI. They have found that functional connectivity at rest included the areas of the VS, the PFC, and occipital and temporal cortices. There are relatively few studies that have examined motivation in situations when a decision is made in the absence of choice. Clithero et al. (2011) have used a task with anticipation of either money or and candies in fMRI. Activation of the NAcc and anterior insula was associated with individual variation in reward choice, and it shows that the NAcc modulates free choice.

### 1.4. The coding of reward in the brain

There is evidence for a modular view of reward value coding in the brain which is indicated by a posterior-anterior organization in the OFC. Sescousse et al. (2010) have used an incentive delay task that has compared brain activation patterns to monetary and erotic rewards. They have shown that the VS, anterior insula, ACC and midbrain, encode the subjective value of rewards. The anterior lateral OFC processes monetary gains, whereas the posterior lateral OFC processes more basic erotic stimuli. Sescousse et al. (2015) have used cues predicting monetary or erotic rewards, with no decision required in fMRI. The VS was activated by both cues and its activity correlated with the motivational value of these rewards. Striatal reward value was activated when choice was not required but it can modulate motivated behavior.

There is little research on the difference between expected information and reward particularly when the payoff and goals are unknown. Filimon et al. (2020) have used a visual probabilistic categorization task in fMRI and they have discovered that expectation of information has activated the left lateral VS. The NAcc, medial PFC and the OFC during information expectation were differently activated than during reward-related processing.

### 1.5. Motivated cognitive control

There is evidence for dissociable neural responses to reward and penalties that are dependent on the psychological context in which they are experienced. Elliott et al. (2000) have used a gambling task with financial rewards and penalties in fMRI. They have shown an activation of the midbrain and VS to financial rewards and hippocampal activation to financial penalties. The globus pallidus, thalamus, and subgenual cingulate were activated in response to high reward. Increased penalties have activated the caudate, insula, and ventral prefrontal cortex.

Decision-making involves a cost-benefit analysis and brain imaging studies have shown that the process activates the VS and dorsal ACC. Schouppe et al. (2014) have used a decision-making task that involved a choice between an easy and a difficult flanker task. The cost-benefit analysis has been shown by activation of the striatum by a cognitively demanding option and not by forced-choice trials. Finally, models of reward implicate cortical-striatal loops and the DA system, with special emphasis on D_2_ receptors in the NAcc. Johansson et al. (2014) scanned participants in PET a [^11^C] raclopride dynamic scan during rewarded and non-rewarded task switching. Rewarded task switching (relative to baseline task switching) has decreased [^11^C] raclopride binding in the NAcc. Decreased NAcc [^11^C] raclopride binding correlated with performance on the task (reaction times). These PET findings provide evidence for striatal DA release during motivated cognitive control, and that DA release in the NAcc predicts the task’s reaction time benefits of reward.

### 1.6. PFC integration of cognitive control

The medial and lateral Pre-Frontal Cortex (PFC) play an important role in motivation reward by integrating motivation and cognitive control during decision-making.

Kouneiher et al. (2009) have reported that the medial and lateral PFC are organized hierarchically from posterior to anterior areas for motivation and behavior selection. Furthermore, functional connectivity analyses have shown that interactions in these regions transmit motivational incentives and regulates top-down control mechanisms. The PFC therefore integrates motivation and cognitive control for the purpose of decision making between short-term or long-term goals (Kouneiher et al., 2009). The PFC can exercise voluntary control by inhibiting activity of the NAcc during situations that require impulse control. Diekhof and Gruber (2010) have assessed the process of deciding between short term and long-term goals in human participants in fMRI. They have found that behavior that favors long-term goals over immediate reward was associated with an interaction between the antero-ventral PFC with the Nacc and VTA. The degree of this interaction predicted behavioral success during pursuit of the long-term goal, and it was associated with trait impulsivity. These findings demonstrate how voluntary action controls immediate desires *via* the inhibition of the PFC (Diekhof and Gruber, 2010). This evidence has important implications for the study of adolescents who are hypersensitive to reward due to an imbalance between reward and cognitive control.

Recent research has suggested that the brain’s reward mechanisms function differently in adolescence. It has been argued that sensitivity to reward is high in adolescence due to an imbalance in development of the striatum and prefrontal cortex, and that this motivation tendency may lead to subsequent substance abuse. It is not clear whether adolescents are involved in risk-taking behavior since they overestimate potential or actual reward. The anticipation, receipt, and omission of reward were investigated in adolescents and young adults in fMRI (Van Leijenhorst et al., 2010). They have reported that in anticipation of uncertain outcomes, the anterior insula was more active in adolescents compared with young adults and that the VS was activated in middle adolescence, whereas young adults showed OFC activation to omitted reward. These findings suggest that adolescents are hypersensitive to reward and that could result in risk-taking behavior.

The monetary incentive delay (MID) task was employed to assess the association between personality traits and reward mechanisms in young adolescents and young adults in fMRI (Joseph et al., 2016). Unlike the predictions, adolescents did not show higher sensitivity to gains than losses compared with adults during either anticipatory or feedback phases. They did show lower sensitivity to incentive magnitude in the meso-limbic circuit during anticipation and feedback stages. This response was mediated by personality, so impulsive or low avoidance adolescents showed greater gain sensitivity and high avoidance adolescents showed high loss sensitivity during cue anticipation. In adults, impulsivity has modulated meso-limbic response and impulsive adults showed reduced magnitude sensitivity during both anticipation and feedback. These findings imply that impulsivity modulates activity of the mesolimbic reward circuit during both adolescence and adulthood but in adolescence, avoidance and approach also affect this activity.

The OFC has an important function during reward anticipation and consummation. Yan et al. (2016) have found that the OFC encoded magnitude and valence (win vs. loss) but not outcome (favorable vs. unfavorable) during reward consummation and that the lateral OFC but not the medial OFC encoded information about loss. The dorsolateral PFC (dlPFC) integrates and transmits signals of reward to the mesolimbic and meso-cortical DA circuits and initiates motivated behavior. Ballard et al. (2011) have studied participants who have anticipated and prepared for opportunities to get reward in fMRI. They have reported that the availability of reward activated the dlPFC which monitored activation of the VTA. The dlPFC therefore integrates and transmits signals to the mesolimbic and meso-cortical DA systems, thus initiating motivated behavior (Ballard et al., 2011).

Although medial frontal brain regions are implicated in valuation of rewards, there are few studies on reward motivation using focal lesions to these areas. Manohar and Husain (2016) have studied patients with isolated, focal damage in ventral-medial PFC. Medial frontal damage was associated with reduced reward effects on saccadic velocity and autonomic (pupil), although few patients showed abnormally strong reward motivation effects. Increased sensitivity to rewards within the lesion group correlated with damage in subgenual ventral-medial PFC areas. Although medial frontal lesions may generally reduce reward sensitivity, damage to key areas paradoxically protects from this effect. Finally, Etzel et al. (2016) have used task switching with random reward incentive and no-incentive trials in fMRI and they have found that information on the task could be elucidated from activation of frontal-parietal regions associated with task control.

### 1.7. Reward motivation activates the motor system

There is further evidence that reward motivation activates the motor system which integrates information about reward value to motivate performance. The executive control mechanisms involved in maintaining a balance between executing and withholding an action were investigated by Lee et al. (2017). They have manipulated prospective rewards in a stop-signal task in fMRI where both the proactive and reactive control were equally emphasized and showed that inhibition activated the anterior caudate (motivation status) and pre-SMA (action). The pre-SMA was activated in response to the need for action control, whereas the right inferior frontal cortex was activated during action inhibition. Galaro et al. (2019) have used a motor incentive motivation task for prospective monetary gains and losses together with motor cortical excitability measurement with transcranial magnetic stimulation (TMS). They have found that prospective gains and losses improved individuals’ performance. Loss aversion predicted behavioral sensitivity to incentive and motor cortical sensitivity to prospective gains. These findings suggest that motor cortical activity integrates information about the subjective value of reward to enhance incentive-motivated performance. Finally, Cho et al. (2013) used the MID task to study adults and youth in fMRI. They have found that the thalamus and insula have modulated activity of the NAcc during incentive processing. They have suggested that in both adults and adolescents, anticipation of gain or loss involves an “alerting” signal from the thalamus that together with interoceptive signals from the insula shapes selection programs in the VS.

### 1.8. Reward motivation affects incentives like food, money, and social reward

Reward motivation also affects executive control mechanisms when an individual estimates future actions based on motivational states. Setton et al. (2019) have compared the effects of hunger and satiety states on participant’s bids on snack foods in fMRI. They have shown that activation of reward and control areas is affected by motivational states like hunger and satiety and that congruence of present and future motivational states affects any predictions about the future.

The motivation to obtain and consume primary rewards such as food is regulated by devaluation procedures but not by secondary rewards such as money. Yang et al. (2021) have investigated devaluation for primary reinforcers like chocolate milk and secondary reinforcers like money in fMRI. Functional near-infrared spectroscopy (fNIRS) measures obtained during the incentive delay paradigm showed that increasing value of secondary reward was linked with increasing anticipatory reward that has activated the lateral OFC, whereas during the consummation phase, the secondary reward activated the medial OFC irrespective of devaluation stage. These findings imply that secondary reinforcers like money can increase incentive motivation with repeated reward.

Dubey et al. (2020) have used behavioral measures of social motivation in order to assess the neural correlates of social and object rewards. They have used the “Choose-a-Movie-CAM” which quantifies the motivation for seeking social rewards including stimulus value and the effort required to obtain it (cost-benefit) in fMRI. They have reported that the precuneus and medial OFC were involved in social choice whereas the ventral and dorsal striatum were involved in object choice. These activations can be seen during the decision phase even before the rewards have been consumed, indicating a transfer of the rewarding value of social stimuli to its cues. The left insula and bilateral inferior occipital gyrus and the inferior parietal lobe were recruited for increasing effort investment. Finally, Brandl et al. (2019) have performed meta-analysis of studies using cognitive reward control of one’s craving for hedonic stimuli, like food, sex, or drugs in fMRI. They have discovered cognitive reward control activation mainly in the supplementary motor area (SMA), dlPFC, and ventrolateral PFC across studies. The cognitive reward control was activated across stimulus types, and it was similar to areas mediating cognitive emotion regulation. Table 1 describes reward motivation studies in fMRI.

**TABLE 1:** Brain imaging studies of reward motivation^1^.

References	Methods	Participants	Task	Main findings
Elliott et al., 2000	fMRI	Nine healthy adults	A gambling task with financial rewards and penalties	An activation of the midbrain and VS to financial rewards and hippocampal activation to financial penalties. Activation of the globus pallidus, thalamus, and subgenual cingulate in response to high reward. Penalty activated the caudate, insula, and ventral PFC.
Ernst et al., 2004	fMRI	Seventeen healthy adults	The wheel of fortune decision making task with probabilistic monetary gains	The selection phase activated regions associated with visual-spatial attention (occipital-parietal pathway), conflict (ACC), manipulation of quantities (parietal cortex), and preparation for action (premotor area). The anticipation phase activated regions associated with reward (VS). High-reward/risk conditions correlated with greater neural response in VS during selection but not during anticipation. The OFC was activated during selection, particularly to high-risk/reward options and during anticipation.
Schott et al., 2008	[^11^C]raclopride in PET	Fourteen young healthy adults (age range 20–25, mean age 22.8 (SD = 1.5)	Monetary incentive delay (MID) task	Activity in the SN, VTA, VS and NAcc in fMRI correlated with dopamine release in the NAcc measured in PET. Dopamine release correlated with activity in the amygdala and the hippocampus.
Locke and Braver, 2008	fMRI	Twenty healthy university students	The Continuous Performance Test (CPT) under three different blocked motivational conditions (reward-incentive, penalty-incentive, and baseline).	Reward activated a right-lateralized network that included parietal and prefrontal cortex. Activation in both reward-related brain regions and frontal-polar cortex was associated with the degree of motivation-induced performance enhancement and to motivation-related personality variables.
Kouneiher et al., 2009	fMRI	Sixteen participants, age range 19–35 years, 8 females and 8 males	A visual response task to task trials and default trials	The medial and lateral PFC were activated by motivation and selection behavior. Interactions in the medial and lateral PFC regulate top-down control mechanisms.
Symmonds et al., 2010	fMRI	Seventeen healthy participants, age range, 22–36 years; 10 females, 7 males	A sequential choice task	Sequential choice activated the cingulate and insula cortices for anticipated risk and the VS and medial OFC for valuation of reward
Sescousse et al., 2010	fMRI	Eighteen healthy participants mean age 24 (SD = 3.3)	An incentive delay task with monetary and erotic rewards	The VS, anterior insula, ACC and midbrain, encode the subjective value of rewards. The anterior lateral OFC, processes monetary gains, the posterior lateral OFC, processes erotic stimuli.
Diekhof and Gruber, 2010	fMRI	18 healthy participants, 10 females, 8 males recruited from an academic environment	A sequential forced choice task	Preference for long-term goals over immediate reward activated the antero-ventral prefrontal cortex (avPFC), the Nacc and the VTA. The interaction between these areas predicted behavioral success during pursuit of the long-term goal and it was associated with trait impulsivity.
Van Leijenhorst et al., 2010	fMRI	Fifteen 18–23 year olds (7 females 8 males; mean age = 20.2 (SD = 1.6), eighteen 14–15 year olds (10 females 8 males; mean age = 15 (SD = 0.7), and seventeen 10–12 year olds (8 females 9 males; mean age 11.6 (SD = 0.8).	A slot machine task measuring the effects of anticipation, receipt, and omission of reward	Anticipation of uncertain outcomes activated the anterior insula in adolescents compared with young adults. The VS was activated in middle adolescence. Young adults showed OFC activation to omitted reward.
Harsay et al., 2011	fMRI	Fourteen healthy right-handed participants, mean age, 22.7 (SD = 2) (10 females, 4 males;	A stopwatch task	Reward anticipation activated the VS and dorsal striatum, and cortical oculomotor regions. Functional connectivity between the caudate nucleus and cortical oculomotor control areas predicted individual differences in the behavioral benefit of reward anticipation.
Clithero et al., 2011	fMRI	65 young adults [32 females, 33 males, mean age 24.7 (SD = 5.9)	An anticipation of monetary and candy rewards	Activation of the NAcc and anterior insula predicted individual variation in relative motivation between rewards. NAcc activation, mediated the effects of anterior insula.
Ballard et al., 2011	fMRI	Twelve participants, age, 23.9 years (SD = 3.8) years; 6 males 6 females.	The monetary incentive delay task (MID)	Reward availability activated the dlPFC which monitored activation of the VTA.
Cho et al., 2013	fMRI	Thirty healthy adults, age range 22–48 years mean age 28.8 (SD = 7.5) 11 males and 19 females), and twenty-four healthy adolescents, age range 10–17 years mean age 14.6 (SD = 2.0) 15 males and 9 females	The monetary incentive delay (MID) task	The thalamus and insula activated the NAcc during incentive processing.
Johansson et al., 2014	[^11^C]raclopride in PET	Thirteen males, age range 35–40 years mean age 38 (SD = 1.7)	Rewarded and non-rewarded task switching	Rewarded task switching, relative to baseline task switching, decreased [^11^C] raclopride binding in NAcc. Decreasing NAcc [^11^C] raclopride binding was correlated with task reaction time measures. Reward-related DA release in the antero-dorsal caudate.
Schouppe et al., 2014	fMRI	Twenty-five right-handed students age range 19–25 years 22 females 3 males	An effort-based decision-making task	Choice-locked activated the striatum when participants chose voluntarily for the more effortful alternative but was de-activated the striatum on forced-choice trials.
Sescousse et al., 2015	fMRI	38 healthy right-handed participants mean age 27.5 years (SD = 6.8)	Single cues predicting monetary or erotic rewards, without making a decision	The ventral striatum and the frontal-parietal network were activated by both cues and its activity correlated with the motivational value of these rewards.
Joseph et al., 2016	fMRI	Fifty-one healthy young adults age range 18–25 years and 27 adolescents age range 11–14 years	The Monetary incentive delay (MID) task	Adolescents did not show higher sensitivity to gains than losses compared with adults during either anticipatory and feedback phases. They did show lower sensitivity to incentive magnitude in the meso-limbic circuit during anticipation and feedback stages. In adults, impulsivity modulated meso-limbic response and impulsive adults showed reduced sensitivity during both anticipation and feedback.
Yan et al., 2016	fMRI	Twenty-three participants (mean age: 19.78, SD = 0.8) (12 males 11 males)	The monetary incentive delay task (MID)	Activation of the OFC encoded win vs. loss status but not outcome (favorable vs. unfavorable) during reward consummation. The lateral OFC encoded loss information. The OFC encoded values in a similar way to the VS or the anterior insula during reward anticipation regardless of motivated response and reward consummation.
Manohar and Husain, 2016	fMRI	22 Patients with focal damage in VMPFC Mean age 49.6 years, (SD = 10.8)	A saccadic task using an auditory incentive cue to quantify reward sensitivity	Reduced reward effects on saccadic velocity and autonomic (pupil). Increased sensitivity to rewards correlated with damage in subgenual vmPFC.
Kasanova et al., 2017	[^18^F] fallypride in PET	16 healthy volunteers	A probabilistic reinforcement learning task	A correlation between reward-related DA release in the putamen, caudate and ventral striatum and performance on the probabilistic rewarding task. Dopamine release in the right caudate and ventral striatum regulated behavior if it was enjoyable.
Etzel et al., 2016	fMRI	Twenty-four young students	Cued task switching	Cue-related activation patterns in frontal-parietal regions correlated with task control. Information trained at baseline had higher decoding accuracy on incentive than non-incentive trials, with decoding improvement mediating reward-related enhancement of behavioral performance.
Lee et al., 2017	fMRI	Eighteen healthy college students, mean age 22 10 males, 8 females	Stop signal task	Inhibition activated the anterior caudate (motivation status) and pre-SMA (action). The pre-SMA was activated to accommodate motivation for action control, the right inferior frontal cortex participated in the execution of action inhibition.
Mori et al., 2019	fMRI	Forty five healthy volunteers	A monetary incentive delay task	Functional connectivity at rest included the VS, the PFC, and occipital and temporal cortices.
Galaro et al., 2019	fMRI	19 participants Mean age 20 years; age range 18–23 years 12 females, 7 males	A motor incentive motivation task	Loss aversion predicted behavioral sensitivity to incentive and motor cortical sensitivity to prospective gains. Motor cortical sensitivity to incentive reward mediated the relationship between subjective preferences for incentive reward and behavioral sensitivity to incentive reward.
Filimon et al., 2020	fMRI	Ten right-handed participants age range 22–33 years; mean age 25.7 years, 6 males 4 females	A visual probabilistic categorization task	The expectation of information activated the left lateral VS. Different activations of the NAcc, medial PFC and the OFC during information expectation compared with reward-related processing.
Dubey et al., 2020	fMRI	Twenty-four healthy adults age range 19–49 Mean age 29.14 years (SD = 8.28) 13 males and 11 females	”Choose-a-Movie-CAM” which quantifies the motivation for seeking social rewards including stimulus value and the effort required to obtain it (cost-benefit)	The precuneus and medial OFC were activated during social choice and the VS and dorsal striatum were activated by object choice. The left insula and bilateral inferior occipital gyrus and the inferior parietal lobe were activated during effort investment.
Yang et al., 2021	Functional near-infrared spectroscopy (fNIRS)	The incentive delay paradigm with fNIRS included 35 healthy male participants, Mean age 21.74 years (SD = 2.02), age range 18–25, Mean BMI = 20.91 (SD = 1.52), range = 18.38–23.67)	Choice reward paradigm and an incentive delay paradigm	Increasing value of the secondary reward (money) was associated with increasing anticipatory activation in the lateral OFC, whereas during the consummation phase, the secondary reinforcer was associated with medial OFC activation irrespective of devaluation stage.

^1^Studies arranged chronologically.

ACC, anterior cingulate cortex; DLPFC, dorsolateral prefrontal cortex; SN, substantia nigra; SMA, supplementary motor area; OFC, orbitofrontal cortex; NAcc, nucleus accumbens; PFC, prefrontal cortex; SPL, superior parietal lobule; VTA, ventral tegmental area; VS, ventral striatum; vmPFC, ventromedial prefrontal cortex.

## 2. Motivation reward association with learning, memory, and attention

### 2.1. Learning

Individuals are motivated to act upon either internal urges or interests or external triggers like money and this process of orientation can affect positive or negative feedback and learning. Linke et al. (2010) have used a probabilistic reversal learning task in order to investigate how motivation regulates learning. Extrinsic motivation correlated with activation of the ACC, amygdala and putamen, whereas intrinsic motivation negatively correlated with activation in these brain regions. These findings indicate that motivational orientation regulates response to reward in the ACC, amygdala, NAcc, and the putamen.

Learning motivation activates different brain regions than motivation to gain monetary reward. Mizuno et al. (2008) have reported that learning motivation correlates with activation of the putamen, whereas monetary reward has also activated the putamen bilaterally, though there was no association between activation and monetary reward.

Murayama et al. (2010) have used a stopwatch task in fMRI and showed that performance-based monetary reward has undermined intrinsic motivation indicated by reduced task engagement and decreased activity in the anterior striatum and PFC areas. It seems that the cortical-basal ganglia reward valuation system integrates extrinsic and intrinsic task reward value. Miura et al. (2017) have used variations of the stopwatch task that manipulated controllability and outcome in fMRI. The stopwatch task with the action-outcome contingency activated greater enjoyment and the VS and midbrain, indicating intrinsic motivation.

Previous studies suggest that external rewards like money and verbal praise can enhance motivation and self-determination. Albrecht et al. (2014) have reported that positive performance feedback activated the anterior striatum and midbrain while monetary rewards were administered. Verbal rewards have activated these regions when participants received success compared to failure feedback. Finally, after the verbal rewards were administered and withdrawn, activity in the lateral PFC has increased.

It is unclear how intrinsic rewards that are associated with a task motivate cognitive control. Huskey et al. (2018) have used a video game and showed that high levels of intrinsic reward, with a balanced task difficulty and individual ability, were associated with increased activation of cognitive control areas (dlPFC), orienting attention (precentral gyrus SPL), attention alert (insula), and reward (putamen). However, a discrepancy between task difficulty and individual ability reduced intrinsic reward and has activated the default mode network, including the insula and the salience network.

Reward motivation and information processing by cues was investigated by Shashidhara and Erez (2021). They have used a cued target detection task together with monetary reward and they have found that reward activated the frontal-parietal network when the cue and subsequent object were presented. However, unexpectedly, reward did not increase discrimination between conditions when context information was provided.

### 2.2. Memory and DA in the striatum

The striatal dopamine reward circuit has dense dopaminergic innervation which is responsible for motor control and higher-order cognitive brain functions. The limbic striatum, that is responsible for reward processing, has been associated with episodic memory. Cervenka et al. (2008) have investigated the association between cognitive performance and striatal dopamine D2- receptor binding with [^11^C] raclopride in PET. Receptor availability in the limbic striatum was related to performance in tests of episodic memory, but not to tests of verbal fluency and general knowledge. By contrast, D_2_ binding in associative and sensory-motor striatum was associated with non-episodic tasks. These findings provide evidence for a functional compartmentalization of human striatum and serve as a starting point for a more detailed investigation of striatal biomarkers in the normal brain as well as in neurodegenerative disorders.

### 2.3. Memory formation

Activity in the VS works together with the frontal-parietal attention network to enhance memory formation. Duan et al. (2020) have shown that both external monetary reward and internal curiosity have enhanced memory performance, without evidence for an interaction between the two. Curiosity activated the VS reward circuit and the frontal-parietal attention circuit to enhance memory formation. The external monetary reward effect on memory was associated with reduced activity in parietal midline regions. Curiosity therefore enhances memory performance by allocation of attention and reward resources whereas monetary reward does so by suppression of task-irrelevant processing.

### 2.4. Memory encoding and consolidation

Memory encoding and consolidation is affected by activation of the hippocampus and the reward circuit. Adcock et al. (2006) have used a monetary incentive encoding task with reward cues for memorizing an upcoming scene in fMRI. Rewarding cues presented before remembered scenes have activated the Nacc, VTA and the hippocampus, and they have been associated with improved declarative memory. Supporting evidence for the role of reward motivation in activation of memory in the hippocampus is provided by Murty and Adcock (2014). They have used a task presenting participants with goal-irrelevant expectancy violations during high or low-reward motivation. Reward motivation has activated the hippocampus in response to declarative memory for expectancy violations and it enhanced anticipatory VTA-cortical-hippocampal interactions. Murty et al. (2016) have exposed participants to unexpected events during reward or punishment in fMRI. The hippocampus was activated by perceptual surprises during reward motivation, whereas surprises have activated the parahippocampal cortex during punishment. These findings indicate that separate parts of the MTL regulate anticipation of reward and avoidance of punishment, affecting learning and handling of surprises in order to make predictions about the environment. Miendlarzewska et al. (2016) have reviewed the evidence of how motivation can prioritize information for memory encoding and consolidation. The evidence suggests that dopamine enhances the formation of declarative memory for reward information and may also control the generalization of reward values. In particular, activity in the hippocampus and in the reward circuit affects decision-making and integration.

### 2.5. Memory recollection

Reward motivation also plays also a role in modulating memory recollection (Elward et al., 2015). Participants have discriminated between studied and unstudied pictures of objects, and they have indicated the identity of the coin paired with the object at study. Correct judgments earned a reward corresponding to the value of the coin, whereas incorrect judgments were penalized. Accurate responses have activated the hippocampus and different striatal sub-regions demonstrated recollection effects, reward effects, and overlap between the two effects. The left angular gyrus and medial prefrontal cortex were additively responsive to source accuracy and reward.

### 2.6. Long-term memory of novelty

Novelty and reward were investigated by using a scene encoding paradigm in fMRI (Bunzeck et al., 2012). The striatum and the SN/VTA have modulated long-term memory activity in the MTL and contextual exploration in the hippocampus. This evidence suggests that long-term memory of novelty stored in the MTL is associated with reward-prediction in the medial OFC, which in turn affects reward responses in the striatum.

### 2.7. Episodic memory retrieval

Reward motivation and emotion have also distinct effects on episodic memory. Shigemune et al. (2010) have reported a study in which subjects were engaged in encoding of photographs during an imaging scan using H_2_^15^O in PET. During encoding, emotional exposure to negative pictures has activated the left amygdala whereas the left OFC was activated during encoding of high reward pictures relative to low reward pictures, and the hippocampus was activated in both conditions. It has been suggested that the hippocampus integrates the effects of emotion in the amygdala and monetary reward in the OFC on encoding of episodic memory (Shigemune et al., 2010). Shigemune et al. (2017) have shown that reward motivation enhanced episodic memory of Japanese words and it has activated the SN/VTA, MTL, dorsomedial prefrontal cortex (dmPFC), and dlPFC during retrieval of memories with high difficulty. Finally, Frank et al. (2019) have found that connectivity between memory and reward regions reflected individual differences in reward modulation of memory, in the AC, OFC, and VS. This evidence suggests that there is a broader set of reward regions regulating memory than considered previously and these relate to individual differences in how reward impacts memory.

### 2.8. Motor imagery and imagery of future events

Reward-related cues presented during motor imagery have activated the VS, the amygdala and the motor cortex, increasing the desire and urge to obtain goals (Mendelsohn et al., 2014). Reward motivation also affects imagery of personal future events. Bulganin and Wittmann (2015) have used a reward learning task in which words have either received a monetary reward or not. They then imagined personal future events based on these and novel words. Reward and imagery of future events have activated the striatum, VTA and the hippocampus. Functional connectivity between these areas was enhanced during imagery of reward and novel words. This study indicates that past motivated reward contributes to our expectation of the future.

### 2.9. Working memory

Motivation can also enhance attention, goal achievement and working memory.

Jimura et al. (2010) showed that individuals who are reward-sensitive improved working memory on trials that were not rewarded which has activated the right lateral prefrontal cortex. Motivation can also be integrated in executive function areas such as the dorsal ACC to compute the expected value of goal-directed cognitive control. Yee et al. (2021) have used a task that quantifies the combined effects of liquid and monetary incentives on cognitive task performance. They have found that monetary incentives adjusted dorsal ACC activation and this adjustment predicted changes in cognitive performance and self-report motivation ratings. The dorsal ACC therefore encoded the incentives in terms of their motivational value, which correlated with task performance.

### 2.10. Attention

Reward and motivation have beneficial effects on accuracy and variability during sustained attention. Locke and Braver (2008) have used the Continuous Performance Test (CPT) under different motivational conditions. They have found that reward was associated with an activation of the parietal and prefrontal cortex. Activation of reward and PFC was associated with motivation-related performance enhancement and personality variables. Esterman et al. (2017) have used the gradual-onset continuous performance task with alternating rewarded and unrewarded blocks. Rewarded blocks have activated brain regions responsible for preparation for upcoming targets, which in turn has improved accuracy. These findings suggest that motivated individuals use task-positive resources proactively during sustained attention. Table 2 describes studies on learning memory and attention in fMRI.

**TABLE 2:** Motivation reward activation of learning, memory and attention in fMRI^1^.

References	Methods	Participants	Task	Main findings
Adcock et al., 2006	fMRI	12 healthy adults age range 18–35 years, 3 female 9 males	A monetary incentive encoding task	In the encoding task, high-reward cues preceding remembered scenes activated the VTA, Nacc and the hippocampus, and their activation predicted superior memory performance. Correlation between the hippocampus and VTA was associated with enhanced long-term memory for the subsequent scene.
Cervenka et al., 2008	[^11^C] raclopride in PET	Sixteen healthy adults age range 41–65 years Mean age 56 (SD = 8) 8 females and 8 males	Episodic memory verbal fluency and general knowledge tests	Receptor availability in limbic striatum was related to performance in tests of episodic memory, but not to tests of verbal fluency and general knowledge. Low correlation between D_2_ binding in associative and sensorimotor striatum with episodic memory, high correlation with the non-episodic tasks.
Mizuno et al., 2008	fMRI	Fourteen healthy college students mean age 22.4 (SD = 1.2), 7 females and 7 males	Monetary reward task	Motivation to learn correlated with bilateral activity in the putamen. A positive correlation between subjective motivation and activity in the putamen. Monetary motivation activated the putamen bilaterally, but activity was not correlated with monetary reward.
Linke et al., 2010	fMRI	33 participants mean age 22.64 (SD = 2.92) age range 19–32) 17 males, 16 females	A probabilistic reversal learning task	Rewarding trials activated the medial OFC and ACC, the amygdala and NAcc. Punishment activated the medial and inferior PFC, the superior parietal cortex and the insula. Extrinsic motivation positively correlated with activation of the ACC, amygdala and putamen, whereas intrinsic motivation negatively correlated with activation in these brain regions.
Murayama et al., 2010	fMRI	Twenty-eight right-handed healthy students, mean age 20.6 (SD = 1.1) 10 male and 18 females	A stop-signal task	Decreased activity in the anterior striatum and the PFC with behavioral undermining effect.
Shigemune et al., 2010	H_2_^15^O imaging in PET	20 male participants age range 20–27 years mean age 21.2 years	An intentional encoding of photographs (emotional, negative or neutral) and monetary reward value, high or low for subsequent successful recognition	During encoding, emotional exposure to negative pictures activated the left amygdala whereas the left OFC was activated during encoding of high reward pictures relative to low reward pictures, and the hippocampus was activated in these two conditions.
Jimura et al., 2010	fMRI	Thirty one healthy participants	Working memory	Highly reward-sensitive individuals exhibited greater improvement of working memory performance in rewarding contexts within the right lateral PFC, exclusively on trials that were not rewarded. Motivation can also be integrated in executive function areas such as the dorsal ACC to compute the expected value of goal-directed cognitive control.
Bunzeck et al., 2012	fMRI	Fourteen participants age range 19–34 years Mean age 22.4 years (SD = 3.8) 5 males and 9 females	A scene encoding paradigm	Reward related long-term memory for the scenes (after 24 h) correlated with activity of the MTL, VS, and SN/VTA. The hippocampus showed the main effect of novelty, the VS showed a main effect of reward, and the medial OFC showed both novelty and reward. The interaction between novelty and reward which is associated with novelty was found in the hippocampus.
Murty and Adcock, 2014	fMRI	Twenty six healthy participants age range: 18–36 years Mean age 24.5 years	A reaction-time task with goal-irrelevant expectancy violations in states of high- or low-reward motivation.	Reward motivation activated the hippocampus in response to declarative memory for expectancy violations. The VTA was connected with medial prefrontal, ventrolateral prefrontal, and visual cortices and it predicted hippocampal activation.
Vassena et al., 2014	fMRI	22 participants Age range 18–24 mean age 20 18 females 8 males	A mental arithmetic task with manipulation of effort, reward and delay in reward delivery	A difficult task with higher reward prospect activated the ACC and the striatum.
Mendelsohn et al., 2014	fMRI	Eighteen healthy, right-handed participants mean age 26.8 years (SD = 3.4), age range 23–36, nine females	The Pavlovian-to-instrumental (PIT) paradigm	Reward-related cues presented during motor imagery activated the VS and amygdala and the motor cortex, increasing the desire and urge to obtain goals.
Albrecht et al., 2014	fMRI	Sixty four participants age range 18–34 years, mean age 24.16 (SD = 3.26) 38 females, 26 males	A monetary and verbal reward task	Positive performance feedback activated the anterior striatum and midbrain while monetary rewards were administered. Verbal rewards activated the anterior striatum and midbrain when participants received success compared to failure feedback. Activity in the lateral PFC was enhanced after the verbal rewards were administered and withdrawn.
Elward et al., 2015	fMRI	Twenty right-handed English-speaking adults age range 18–29	A source memory procedure	Source accuracy has activated the hippocampus. Different striatal sub-regions demonstrated exclusive recollection effects, exclusive reward effects, and overlap between the two effects. The left AG and medial PFC were activated in response to source accuracy and reward.
Bulganin and Wittmann, 2015	fMRI	21 participants mean age 24.3 years (SD = 3.4) 8 males 13 females	A reward learning task	Reward and novelty-based imagery of future events correlated with activation of the motivational system (VS and VTA) and the hippocampus, and functional connectivity between these areas increased during imagination of events based on reward-associated and novel words.
Murty et al., 2016	fMRI	Forty nine participants age range 18–36 Mean age 25	A task recording of mnemonic encoding of surprising events	During reward motivation, perceptual surprises activated the hippocampus, whereas during punishment motivation surprises activated the PG. Reward motivation facilitated hippocampal coupling with vmPFC, whereas punishment motivation facilitated PG coupling with the OFC.
Shigemune et al., 2017	fMRI	Thirty-three right-handed undergraduate and graduate students	Episodic retrieval	Reward activated the SN and VTA), MTL, dorsomedial PFC, and dlPFC during retrieval of memories with high difficulty. Reward-related enhancement of functional connectivity between the SN/VTA and MTL and between the SN/VTA and dorsomedial PFC during the retrieval of memories with high difficulty correlated with reward-related increases of retrieval accuracy and subjective motivation.
Esterman et al., 2017	fMRI	Sixteen participants mean age = 22 years age range 19–29 10 males 6 females	A gradual-onset continuous performance task with alternating motivated (rewarded) and unmotivated (unrewarded) blocks	During motivated blocks, there was an activation in preparation for upcoming targets, in dorsal attention, ventral attention, and frontal-parietal and bilateral PG networks. During unmotivated blocks, no such advanced preparation was observed.
Miura et al., 2017	fMRI	Thirty-six healthy volunteers Age range: 18–36 years Mean age 22 (SD = 3) 18 males and 18 females	A stopwatch task	The stopwatch task with the action-outcome contingency activated the VS and midbrain, indicating intrinsic motivation. Cost-benefit evaluation activated the dorsal ACC (dACC) and the striatum, indicating the discounting effect of effort on reward.
Huskey et al., 2018	fMRI	Eighteen students. Mean age 22.83 (SD = 4.02) 14 females 4 males	A naturalistic and open-source video game stimulus	Intrinsic reward activated cognitive control areas (dlPFC), orienting attention (precentral gyrus SPL), attention alert (insula), and reward (putamen). Low intrinsic reward increased activity within the default mode network, including the insula and the salience network.
Setton et al., 2019	fMRI	Twenty-five healthy participants Mean age 22.52 (SD = 2.79) age range 18–30 years 15 female, 10 males	A task requiring to place bids on snack foods	Projection bias, the difference between bids during incongruent prospection (hungry to satiated, session one) and realization (satiated, session two), negatively associated with thalamic and insular activity. Bias was associated with activation of the VS.
Frank et al., 2019	fMRI	24 participants, age range 18–31 mean age 22 18 females 6 males	A monetary incentive encoding task	Connectivity between memory and reward regions reflected individual differences in reward modulation of memory, in the AC, OFC, and VS.
Duan et al., 2020	fMRI	Thirty-five healthy right-handed young participants Mean age 22.9 years, (SD = 3.13) years 22 females 13 males	Presentation of trivia questions with monetary reward	Curiosity-driven activity in the VS reward circuit worked together with the frontal-parietal attention circuit to enhance memory formation. The external monetary reward effect on memory was associated with reduced activity in parietal midline regions.
Yee et al., 2021	fMRI	46 participants age range 18–38 years mean age 25.4 years (SD = 4.9) 22 females 24 males	Monetary incentive task and cognitive performance	Monetary incentives was encoded in the dorsal ACC and this encoding predicted changes in cognitive performance and self-report motivation ratings.
Shashidhara and Erez, 2021	fMRI	24 participants age range 18–40 years mean age 25 years 13 females 11 males	A cued target detection task	Reward activated the frontal-parietal network when the cue and subsequent object were presented. Reward did not increase discrimination between conditions when context information was provided.

^1^Studies arranged chronologically.

AG, angular gyrus; ACC, anterior cingulate cortex; DLPFC, dorsolateral prefrontal cortex; MTL, medial temporal lobe; Nacc, nucleus accumbens; OFC, orbitofrontal cortex; PG, parahippocampal gyrus; SN, substantia nigra; PFC, prefrontal cortex; VS, ventral striatum; VTA, ventral tegmental area; vmPFC, ventromedial prefrontal cortex.

## 3. Discussion

The brain’s meso-limbic dopamine reward circuitry is known to control reward-motivated behavior in humans. There is evidence that reward motivation is associated with activation of the human reward system [NAcc and the putamen, anterior cingulate cortex (ACC) and the amygdala], it is regulated by areas in the PFC (medial and lateral Pre Frontal Cortex (PFC), the dlPFC, it is modulated by the thalamus, and insula and may activate action plans in the motor system (SMA).

### 3.1. The monetary incentive delay task (DMRT)

One of the most common methods of assessing reward motivation is the monetary incentive delay task (DMRT) and there were nine meta-analyses of this paradigm.

The monetary incentive delay (MID) task in fMRI by Knutson and Greer (2008) requires participants to perform on a reaction time task in which they press a key in response to a white square. The white square is preceded by a circle which is a cue that a successful performance will result with winning money or by a triangle which is a cue that indicates that successful performance will not be rewarded. Feedback about whether money has been won is given visually immediately after the response. Participants are trained until they succeed in two-thirds of the trials. Afterward, they perform the same task in the fMRI scanner. Event-related fMRI is used to measure brain activity during reward anticipation and receiving feedback.

In an initial meta-analysis of studies using the Monetary Incentive Delay (MID) task, Knutson and Greer (2008) have found that reward anticipation has activated the medial frontal gyrus, the NAcc, the anterior insula, the putamen and the thalamus. More recently, Oldham et al. (2018) in a meta-analysis of studies using the MID task has found that reward anticipation has activated the VS and dorsal striatum, the thalamus and amygdala, the midbrain, the insula, the premotor cortex and supplementary motor area, the occipital cortex and the cuneus. Unlike Knutson and Greer’s (2008) meta-analysis, the medial frontal cortex was not activated. Reward delivery activated the VS and the amygdala, medial frontal, OFC and the posterior cingulate cortex. Wilson et al. (2018) had used data from group maps provided by authors and they have found more extensive activation of cortical regions compared with Oldham et al. (2018). Other meta-analyses have used a broad including a wide range of paradigms of anticipation and/or delivery of monetary reward as well as natural reward (Liu et al., 2011; Diekhof et al., 2012; Bartra et al., 2013). Liu et al. (2011) has conducted a meta-analysis on 142 neuroimaging studies and they have found that reward-related decision-making tasks have activated the NAcc, caudate, putamen, thalamus, OFC, anterior insula, ACC and posterior cingulate cortex (PCC), the inferior parietal lobe and the PFC. The NAcc was activated by both positive and negative rewards during anticipation, outcome, and evaluation of reward. The medial OFC and PCC were activated in response to positive rewards, whereas the ACC, anterior insula, and lateral PFC were activated in response to negative rewards. A meta-analysis on the role of reward prediction and its consumption by Diekhof et al. (2012) has found that the VS plays a role in reward anticipation and consumption and may also be sensitive to different reward magnitudes, while the medial OFC and VMPFC process the magnitude during reward receipt. Bartra et al. (2013) have performed a meta-analysis of 206 studies investigating subjective value (SV) of choice alternatives. They have reported neural networks a subjective valuation system of decision making including the dorsal and posterior striatum, dorsomedial PFC, anterior insula and the thalamus. Dugré et al. (2018) have conducted a meta-analysis of 35 studies of brain activations during anticipation and monetary loss. In both anticipation and loss, participants showed activations of the striatum, (anterior) insula, and anterior cingulate gyrus relative to loss. Loss anticipation activated the ventral-lateral PFC whereas loss receipt has activated the medial PFC. Finally, a meta-analysis of 45 studies of reward anticipation has shown activations in the VS, the middle cingulate cortex, supplementary motor area and the insula (Jauhar et al., 2021). This meta-analysis has indicated that monetary reward anticipation and delivery activate the VS but not the dorsal striatum and are associated with different patterns of cortical activation. The studies shown so far indicate that there are variations in activation depending on the anticipation of win or loss and the method of image-analysis in the assessment of reward (see Jauhar et al., 2021 for review).

Alterations in reward motivation are important for the development of psychiatric disorders particularly during adolescence (Fairchild, 2011). Psychiatric conditions involve alterations in motivational processes or disturbances in reward processing that entail hypersensitivity or hyposensitivity to reward at different processing stages such as reward anticipation or receipt. Externalizing disorders and substance use disorders, are related to reduced activity in the motivation reward circuit during anticipation of reward, whereas enhanced responses to rewarding outcomes and reduced sensitivity to punishment is associated with externalizing disorders such as conduct disorder or oppositional defiant disorder. Adolescents with conduct disorder and comorbid substance dependence who performed on the Iowa Gambling Task showed impaired reward-related decision making (Schutter et al., 2010). In contrast, depression is associated with impaired striatal responses to rewarding outcomes. Depressed adolescents who performed on a behavioral decision-making task involving varying probability and magnitude of reward failed to distinguish between options involving small or large possible reward during conditions involving a high probability of winning. These faulty reward choices at the age of 11 predicted depressive symptoms a year later (Forbes et al., 2007). These results show an attenuation of reward seeking in depression or at those with high risk for developing depression. A meta-analytic review of fMRI studies using the MID task to asses reward anticipation in patients with ADHD has shown VS hypo-responsiveness in ADHD during reward anticipation and the opposite in healthy volunteers (impulsivity-scores positively correlated with VS activation during reward processing) (Plichta and Scheres, 2014). These findings indicate impaired reward anticipation mechanisms in ADHD. The following section will describe the clinical implications of these studies for drug abuse, mental and neurological disorders.

### 3.2. Implications for drug abuse mental and neurological disorders

The research so far has implications for understanding drug abuse and other mental and neurological disorders. It has been suggested that when there is a dysfunction in the brain reward system, which could be caused by certain genetic variants of DA, it could result in high risk for multiple addictive, impulsive and compulsive behavioral disorders such as alcohol and drug use disorders, pathological gambling, compulsive sexual behavior, ADHD, Tourette’s Syndrome, conduct disorder and antisocial behavior (Blum et al., 2000). Psychostimulant drugs like cocaine and amphetamines activate the meso-cortico-limbic system by increasing dopamine release within the NAcc resulting in long-term changes and dysregulation of the meso-limbic dopamine reward circuit (Koob and Volkow, 2010). Recent studies have also shown that natural rewards like computer games are associated with DA release to a similar magnitude as psychostimulants (Koepp et al., 1998; Weinstein, 2010). Brain imaging studies have shown that Internet and Gaming Disorder (IGD) is like other addictions by showing activation in brain regions mediating reward, reduced impulse control and impaired decision making; and reduced functional connectivity in areas responsible for cognitive control, executive function, motivation, and reward (Weinstein et al., 2017; Weinstein and Lejoyeux, 2020). Exposure to gambling cues has activated areas of the DA reward system like drugs of abuse (Potenza et al., 2003; Crockford et al., 2005). Exposure to explicit sexual stimuli has activated these areas in individuals with compulsive sexual behavior disorder CSBD (Voon et al., 2014). It has been suggested that disruption of the dopamine reward system is associated with motivation deficits in ADHD adults, which may contribute to attention deficits and supports the use of therapeutic interventions to enhance motivation in ADHD (Volkow et al., 2011). Furthermore, reduced reward function is a diagnostic criterion for depression, and Anhedonia which is a diminished pleasure and or decreased reactivity to pleasurable stimuli is a core feature of depression that frequently persists after treatment (Admon and Pizzagalli, 2015).

### 3.3. Motivation reward, learning, memory, and attention

There are reciprocal relationships between motivation reward, learning, memory and attention. Memory formation is enhanced by VS and frontal-parietal attention networks activation whereas memory encoding and consolidation is modulated by activity in the hippocampus and reward areas. The striatum is also associated with long-term memory of novelty coded in the MTL and reward-predicting properties in the OFC. Long-term memory and contextual exploration in the hippocampus are modulated by parts of the reward circuit (VS, SN and VTA). Reward motivation and emotion have also effects on episodic memory, attention and working memory relying on a broad network of connectivity consisting of the ACC, OFC, and VS.

Since reward is important to learning and memory processes such as encoding and consolidation of memory, there are important implications for drug and behavioral addictions. It is known that memories are not permanent and there has been an effort lately to use reconsolidation in order to eliminate or reduce the impact of bad memories in order to treat mental health disorders like trauma and drug addiction. Although reconsolidation helps to keep memories “alive,” amnesic medications during the reconsolidation period can habituate or weaken bad memories. Early proof-of-principle studies have established reconsolidation as a potential therapeutic strategy for relapse prevention in drug addiction by using pharmacological agents operating on DA receptors or glucocorticoid receptors or others, although there are no definite results of these studies yet (Sorg, 2012; Exton-McGuinness and Milton, 2018). Rafei et al. (2021) have also proposed a novel framework that combines different cognitive interventions including cue-exposure, memory reconsolidation, and episodic future thinking, in order to reshape maladaptive drug-related memories toward more adaptive memories to support addiction recovery.

### 3.4. Motivation reward pharmacological effects and genetics of dopamine

Genetics may also play an important role in modulating motivation reward, and DA has been hypothesized to provide the basis for the interaction between motivational and cognitive control. Studies have used a rewarding guessing task (Yacubian et al., 2007; Forbes et al., 2009) and the MID task (Dreher et al., 2009) in fMRI have investigated the association between the DA transporter DAT_1_ gene (SLC6A3) and reward anticipation. Increased VS activity was linked with a DAT_1_ 9-repeat Variable Number Tandem Repeat (VNTR), related to lower synaptic discharge of DA. Striatal activity was also linked with a DRD_4_ VNTR (postsynaptic inhibition) and a DRD_2_ -141C deletion (inhibitory DRD_2_ receptors). Aarts et al. (2010) have used a pre-cued switching task in fMRI and have also found that carriers of the 9-repeat 9R allele which is associated with high striatal DA turnover, showed greater activity in the dorsal-medial striatum during reward anticipation than homozygotes for the 10-repeat allele, replicating the previous genetic imaging studies. In addition, variations of the catechol-O-methyltransferase (COMT) Val/Met-polymorphism were associated with PFC activation during anticipation of reward. Met/Met carriers of COMT showed increased tonic DA-levels during performance of the task (Yacubian et al., 2007; Dreher et al., 2009). The studies reviewed so far have investigated the link between brain regions and single selected genes. However, reward motivation is a complex neurobiological mechanism that requires an approach studying multiple genes performed by a Genome Wide Association Study (GWAS) (Loth et al., 2011). Furthermore, dopaminergic medication may have different effects on the different stages of drug use such as anticipation and consummation (Webber et al., 2021). The relationship between DA and reward function appears unlikely to be linear and the ability to investigate the effects of DA medications depends on the behavioral and brain-imaging measures that have been utilized. Finally, DA activity is modulated by DA dopamine synthesis and release, re-uptake, receptor activity and metabolism, as well as by other neurotransmitters like opioids, serotonin 5-HT, acetylcholine, gamma-aminobutyric acid (GABA), brain-derived neurotrophic factor (BDNF) and endocannabinoids (Koob and Volkow, 2010).

### 3.5. Reward motivation and sex differences

Very few studies have investigated sex differences in reward motivation using brain imaging techniques. A study has assessed striatal DA response to unpredictable monetary rewards on a “slot-machine” task by using [^11^C] raclopride in PET (Martin-Soelch et al., 2011). They have unexpectedly found a bilateral reduction in binding potential in women during the reward condition while in men the reduction was only in the right ventral striatum. This evidence suggests that DA release in response to unpredictable reward is lateralized in the human ventral striatum, particularly in males. Another study by Alarcón et al. (2017) has examined sex differences during a risky decision-making task in fMRI. Male adolescents made more risky decisions during the task’s performance, and they showed increased NAcc activation in fMRI relative to female adolescents, that was not mediated by sex hormones. The results support previous evidence that increased motivation and salience of reinforcers is associated with robust striatal response and hence reward sensitivity is affected by sex and psychosocial factors.

## 4. Conclusion

The studies reviewed so far describe brain mechanisms of reward motivation in humans including anticipation and consummation of reward. Regions in the dopamine reward circuit including the striatum together with areas in the PFC, insula and thalamus serve to regulate reward motivation. The reward motivation circuit also interacts or facilitates a variety of cognitive function abilities like attention, memory including working memory and decision-making. These studies improve our understanding of human processes of reward in healthy populations, but they also provide insights in deficient reward mechanisms and their manifestation in clinical conditions like drug addiction, behavioral addictions and neurological disorders like ADHD. There is little research on the genetic mechanisms underlying reward motivation and sex differences in reward motivation. Future studies would be able to fill the gaps in our current knowledge of this important topic.

## Author contributions

The author confirms being the sole contributor of this work and has approved it for publication.
